# Central Latin America: Two decades of challenges in neglected tropical disease control

**DOI:** 10.1371/journal.pntd.0007962

**Published:** 2020-03-26

**Authors:** Peter J. Hotez, Ashish Damania, Maria Elena Bottazzi

**Affiliations:** 1 Departments of Pediatrics and Molecular Virology & Microbiology, Texas Children’s Hospital Center for Vaccine Development, National School of Tropical Medicine, Baylor College of Medicine, Houston, Texas, United States of America; 2 Hagler Institute for Advanced Study at Texas A&M University, College Station, Texas, United States of America; 3 Department of Biology, Baylor University, Waco, Texas, United States of America; 4 James A Baker III Institute of Public Policy, Rice University, Houston, Texas, United States of America; 5 Scowcroft Institute of International Affairs, Bush School of Government and Public Service, Texas A&M University, College Station, Texas, United States of America; 6 Department of Pediatrics, Texas Children’s Hospital Center for Vaccine Development, National School of Tropical Medicine, Baylor College of Medicine, Houston, Texas, United States of America; Brock University, CANADA

Together, the Central Latin American (CLA) region comprising Mexico, the countries of Central America excluding Belize (Guatemala, Honduras, El Salvador, Nicaragua, and Costa Rica), Panama, Colombia, and Venezuela represent a large and geographically diverse region with varied economies and of roughly 250 million people (Fig 1) [1], a population size approximating its United States neighbor to the North.

**Fig 1:**
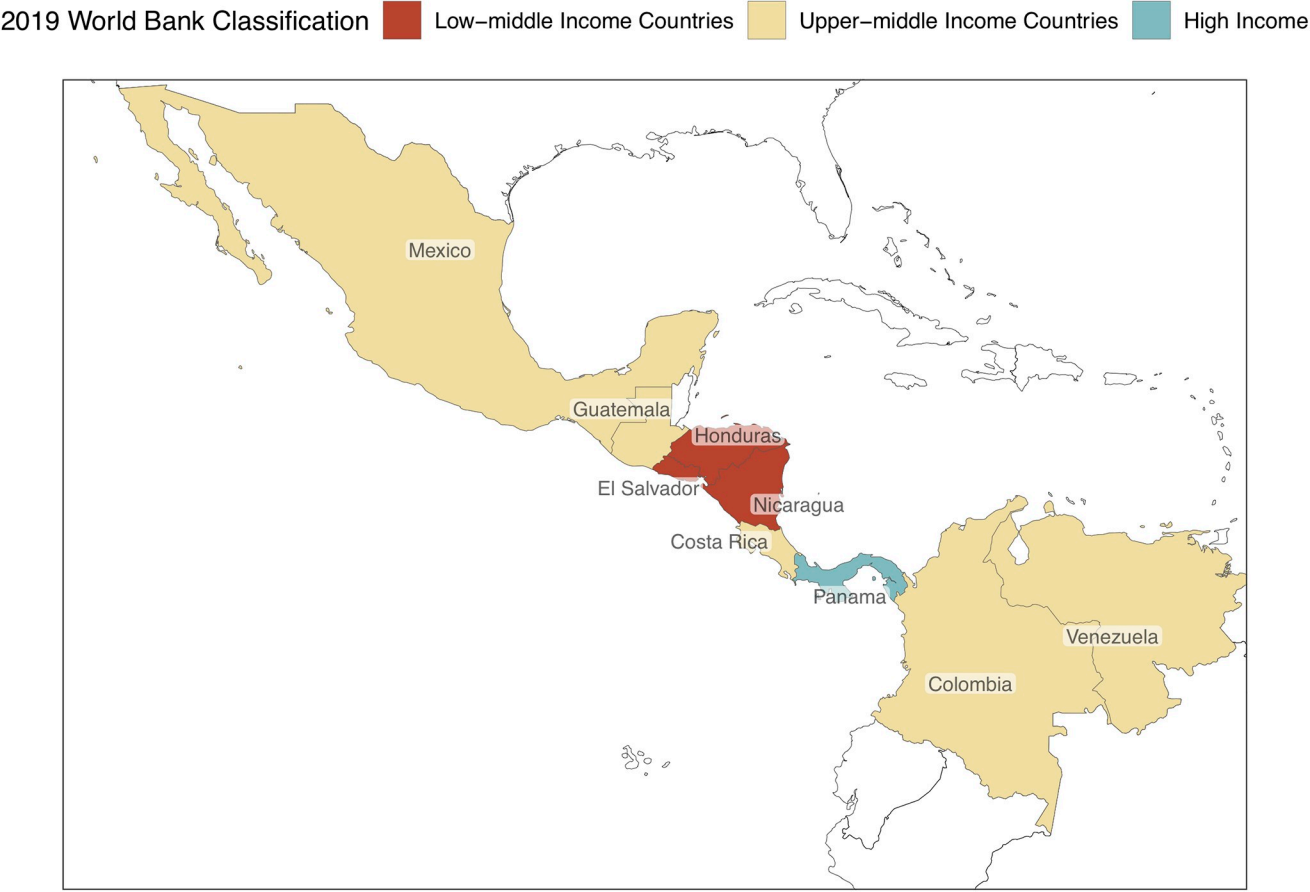
The CLA countries. Nine countries are considered part of this region. The low-middle income countries are red, the upper-middle income countries are tan, and the high-income country is green-blue [4]. Among these countries, Guatemala, Honduras, and El Salvador are also referred to as the countries of the Northern Triangle [2]. In addition, the Pacific Coast of the southern states of Mexico up to Panama compose the CADC [3]. CADC, Central American Dry Corridor.

Since the start of the 21st Century, the CLA region has faced considerable challenges, including prolonged droughts with intermittent and extreme floods due to climate change; violence and political instability linked to the drug trade; political, socioeconomic, and food insecurity from agricultural declines; human displacements; urbanization; and even the marginalization of large indigenous populations [2, 3]. Here, we provide an overview of the findings from the Global Burden of Disease (GBD) Study from the years 2000 and 2017 highlighting the gains or losses in neglected tropical disease (NTD) and malaria disease control in the CLA countries and progress towards the targets of the Global Goals (also known as the Sustainable Development Goals). Furthermore, we provide a perspective of the key physical and social determinants in the CLA region that now and in the future could continue to undermine progress in disease control and elimination efforts.

Table 1 summarizes the overall progress in the increase or reduction of the prevalence or incidence for the control or elimination of NTDs and malaria in the CLA region [5].

**Table 1:** Progress in the Control of NTDs and Malaria in Central Latin America (data from Ref [5]).

Rank	Disease	Prevalence or Incidence in 2000	Prevalence or Incidence in 2017	Change^c^ between 2000 and 2017
1	Trichuriasis	36.32 million^a^	13.45 million^a^	-63%
2	Ascariasis	35.47 million^a^	29.78 million^a^	-16%
3	Hookworm	3.52 million^a^	3.37 million^a^	-04%
4	Chagas disease	1.33 million^a^	1.54 million^a^	+16%
5	Dengue	0.92 million^b^	3.57 million^b^	+288%
6	Malaria	0.84 million^b^	0.71 million^b^	-15%
7	Cysticercosis	0.61 million^a^	0.88 million^a^	+44%
8	Onchocerciasis	0.15 million^a^	<10,000^a^	-96%
9	Leishmaniasis	0.04 million^b^	0.06 million^b^	+50%
10	Zika virus infection	0^b^	1.24 million^b^	Newly Emerged

^a^Prevalence

^b^Incidence

^c^Percent change based on rounded values

NTD, neglected tropical disease

Overall, the GBD 2017 tells a sobering story about progress in controlling malaria and NTDs. Since the launch of the Millennium Development Goals (MDGs) in 2000, there have been enormous successes in reducing the prevalence and disease burdens of malaria and the NTDs in many regions of Africa and Asia [6]. However, within the CLA region with the exception of nearly eliminating onchocerciasis (river blindness), there have been few overall impressive public health gains regarding the NTDs. For example, while we have seen improvements across the region (with the exception of Venezuela) in the control of malaria, trichuriasis, and (at some level) ascariasis, the prevalence of hookworm diseases has only slightly decreased. Furthermore, the prevalence of Chagas disease and cysticercosis in the region has increased. Most dramatically, dengue and leishmaniasis have shown a steep rise in incident cases, and Zika virus infection has newly emerged. A key point, therefore, is that the CLA region seems to be stalled in its progress to control these diseases and continues to be a global “hotspot” for NTDs.

As shown in Fig 2, a more in-depth analysis by country reveals substantial heterogeneity in public health gains and losses across the CLA region. For example, Mexico and Colombia—the two largest CLA nations by population—have made substantial progress in malaria and soil-transmitted helminth control, whereas Chagas disease and dengue continue to rise. Leishmaniasis also increased substantially in Colombia. Overall, the worst performing countries in the region were Venezuela, followed by the three so-called Northern Triangle countries of Guatemala, El Salvador, and Honduras [2].

**Fig 2:**
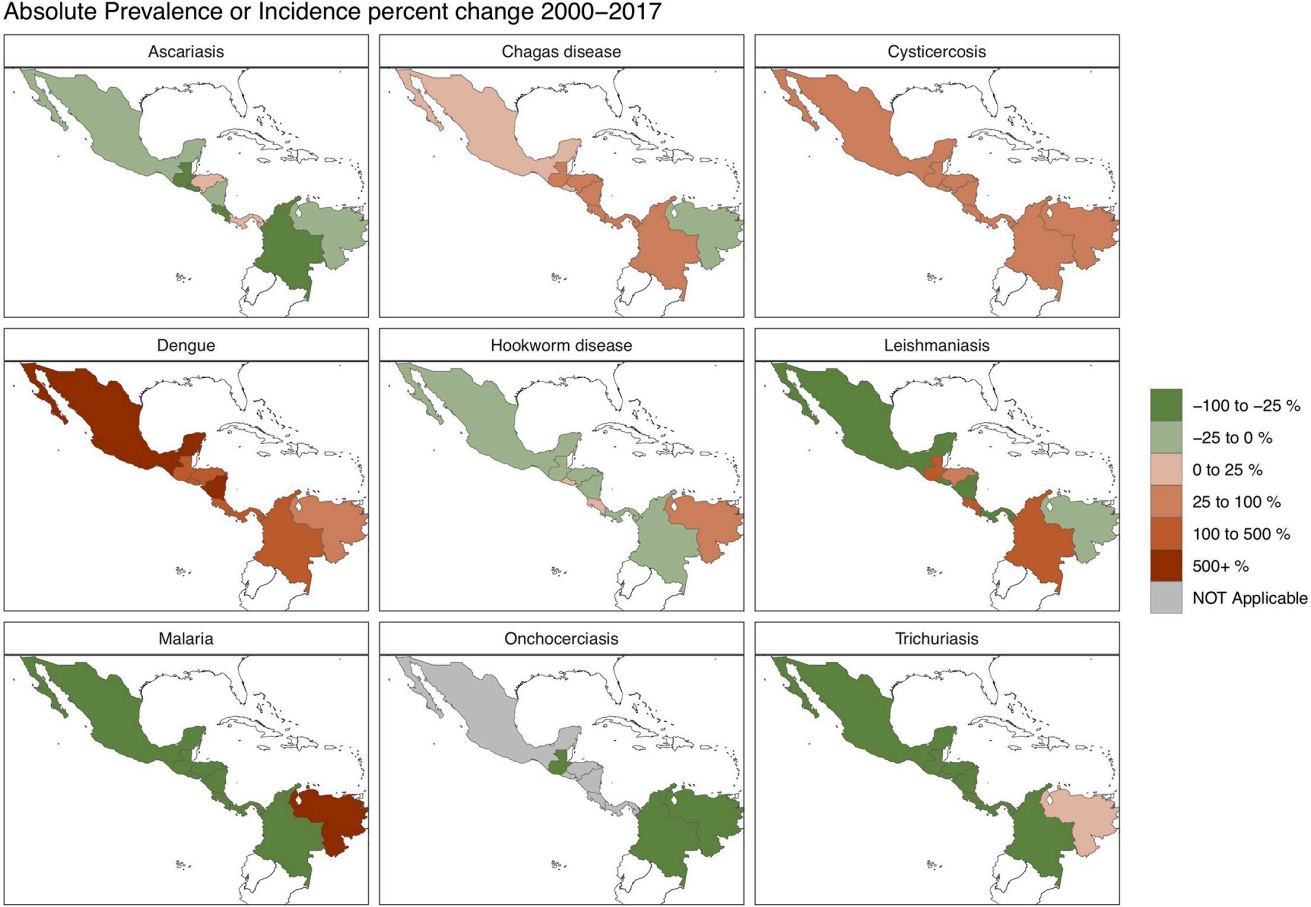
The incidence or prevalence of NTDs and Malaria in the CLA countries. CLA, Central Latin American; NTD, neglected tropical diseases.

The underlying factors behind these observations still require investigation, but there are some key social and physical determinants to consider, especially in some of the underperforming areas.

## Shifts in the illegal drug trade and the rise of the Northern Triangle

Chris Beyrer and his colleagues have pointed out how during the 1990s and 2000s, internal armed conflicts, guerilla movements, kidnappings, and internal displacements linked to cocaine production and trafficking largely halted public health, vector control, and case-detection and treatment activities in Colombia [7]. As a result, there was a sharp rise in malaria, Chagas disease, and leishmaniasis cases [7]. As we moved into the 2010s, drug smuggling routes shifted away from the Caribbean and Florida towards Central America, with major routes operating through the Northern Triangle nations of Guatemala, El Salvador, and Honduras [8]. Briefly, the three countries in the Northern Triangle each face recent and dramatic increases in widespread criminal activity (including organized crime, extortion, forced recruitment into criminal groups, sexual and gender-based violence, and violence against children) as well as economic hardship [2]. New drug routes through the Northern Triangle help to fuel transnational gangs such as Mara Salvatrucha (MS-13) [8]. The rise in violence in this region, together with failed economic policies leading to some of the largest income disparities in the Americas, generated large population displacements now producing large migrant human caravans through Guatemala and Mexico [9]. The public health impact of this political instability and its role in stimulating the rise of NTDs and vector-borne disease in the Northern Triangle remains largely unstudied. However, the impact can be devastating as seen from the spring and summer of 2019 dramatic uptick in dengue cases and severe dengue that was observed in the Northern Triangle countries, especially Honduras and in Nicaragua [10]. Chagas disease, leishmaniasis, and even many of the helminth infections actually increased over the last two decades, so overall within the CLA region, the Northern Triangle countries have made some of the least progress in NTD control, possibly second only to Venezuela.

## Political instability and socioeconomic collapse

An equally urgent economic and humanitarian crisis has unfolded in Venezuela over the last decade. Specifically, the Bolivarian revolution, steep drops in oil revenue, and rise in illegal mining have effectively shut down many aspects of Venezuela’s public health system, with a disproportionate impact on vector-borne tropical diseases and interruption of control activities [11]. Among the prominent aspects is a greater than 400% increase in malaria cases in Venezuela since 2000, together with significant rises in Chagas disease, leishmaniasis, and dengue and the emergence of chikungunya and Zika virus infections [11, 12]. Schistosomiasis increases have also been noted [12]. Similar to the migrant caravans in Honduras, the socioeconomic collapse of Venezuela has fueled a massive population exodus into Brazil, Colombia, and Ecuador, potentially spreading vector-borne diseases.

## The Central American Dry Corridor and climate change

Sociopolitical and economic breakdowns in Venezuela and surrounding countries or in the Northern Triangle of the CLA region are not occurring in isolation. Regarding the Northern Triangle, five years of drought (often interspersed with drenching rains)—in an area that extends from southern Mexico and Guatemala through Honduras and El Salvador and possibly extending as far down as Panama—has produced a Central American “dry corridor” that has adversely affected agriculture in the region, promoting food insecurity, malnutrition, urbanization, and human displacements [13]. The dry corridor is believed to have developed and expanded due to Central America’s disproportionate vulnerability to climate change [3], which can also promote susceptibility to vector-borne tropical diseases, particularly for illnesses transmitted by urbanized *Aedes aegypti* mosquitoes. It’s been further noted that a mysterious form of kidney failure known as Mesoamerican nephropathy has emerged in the dry corridor, where it is sometimes known as global warming nephropathy [14]. Outside of Central America, severe drought from climate change similarly affects Venezuela [15].

## Marginalization of indigenous populations

Indigenous populations are at disproportionate risk to NTDs due to lack of healthcare, extreme poverty, and the fact that their native lands are often exploited for mining and other causes of environment degradation. Such situations are now unfolding among the Yanomami indigenous people in the Amazon region of Venezuela and Brazil, the Wayuu in La Guajira in Colombia near the Venezuela border, and possibly others [16].

## Concluding comments

**Fig 3:**
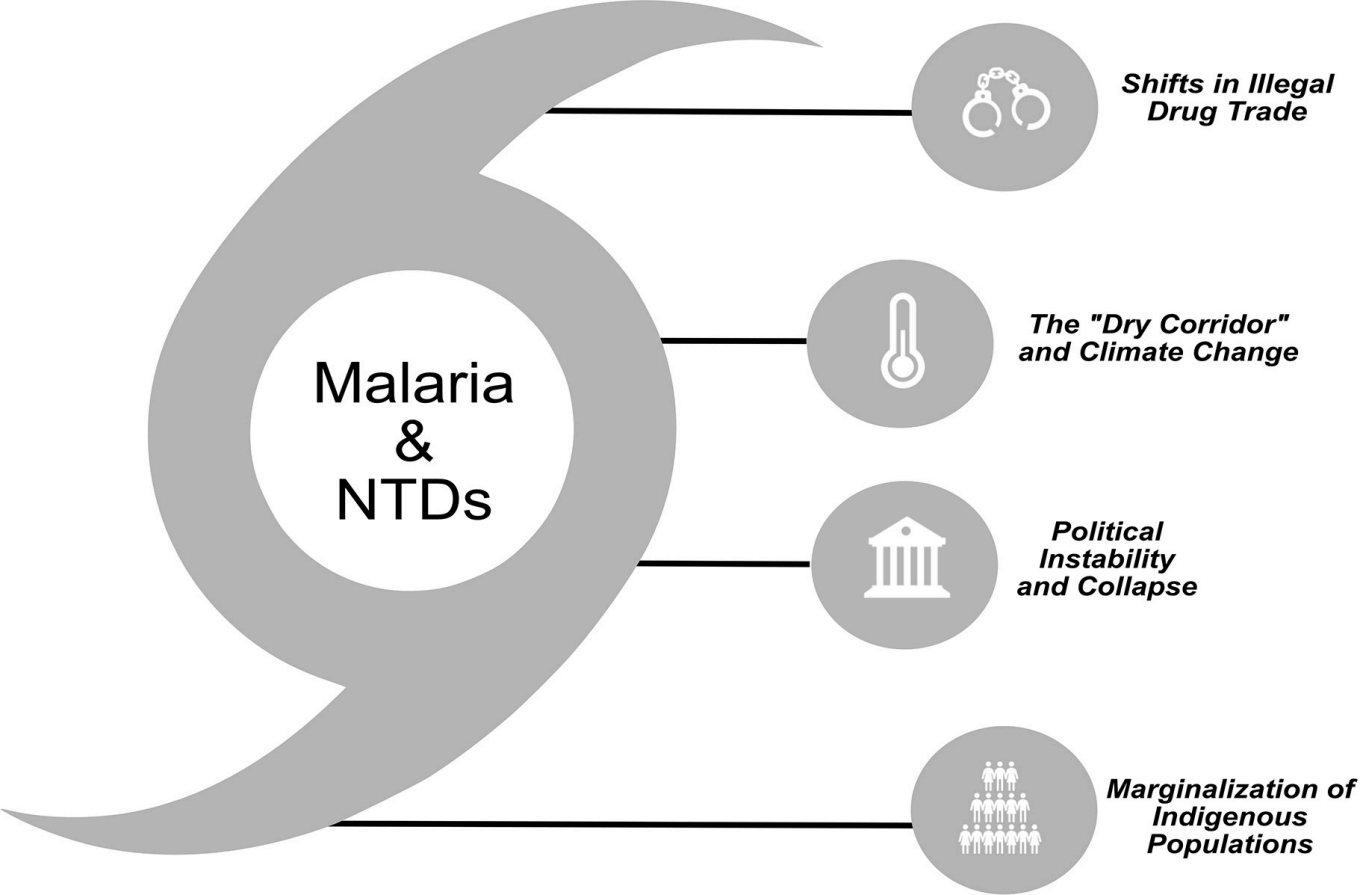
The key vulnerability points and challenges that influence and stall the progress of neglected tropical disease control in the CLA region. CLA, Central Latin American; NTD, neglected tropical diseases.

Together, the countries of the CLA region contain several areas of “perfect storm” conditions (areas highly vulnerable to NTDs, especially vector-borne illnesses). The vulnerability stems of the combination of key physical and social determinants acting together, often synergistically. (Fig 3).

The decline in health systems resulting from these vulnerability points likely account for the minimal public health gains and reductions in NTDs seen over the last two decades. However, it should be noted that progress has been achieved in some areas. For example, Mexico has achieved great progress in terms of the control of malaria, leishmaniasis, and soil-transmitted helminth infections while eliminating onchocerciasis over this period, although Chagas disease and cysticercosis continue to rise and the incidence of dengue has increased dramatically. Similarly, Colombia has made important public health gains in malaria and soil-transmitted helminth control these past two decades, although cases of Chagas disease, leishmaniasis, and dengue continue to go up. Both Venezuela and the Northern Triangle countries continue to have serious challenges leading to—in some cases—tragic increases in their NTDs and, in the case of Venezuela, even a steep rise in malaria. Therefore, additional research is urgently required to understand the marked heterogeneity in NTD and malaria control across the CLA region. Doing so will help identify potential solutions to pinpoint which interventions are effective or which new global heath technologies will need to be developed.

Agencies such as the Organization of American States (OAS) and the Pan American Health Organization could have an important role in convening experts for this purpose. One aspect, for instance, that could accelerate research and development of new or improved global health technologies is building local pharmaceutical development capacity in the CLA region, potentially in Mexico, Panama, and/or Colombia where some infrastructure for biotechnology already exists. This would allow for future self-reliance, sustainability, and economic strengthening while developing and testing new or better drugs, vaccines, and diagnostics specifically designed for the major neglected diseases impacting this troubled part of the world. Nevertheless, getting to the root social and physical causes of the CLA region’s vulnerability points will be paramount for long-term and sustainable solutions to its health system collapse and disease emergence.
